# Cancer mortality and quantitative oil production in the Amazon region of Ecuador, 1990–2010

**DOI:** 10.1007/s10552-013-0308-8

**Published:** 2013-11-30

**Authors:** Suresh H. Moolgavkar, Ellen T. Chang, Heather Watson, Edmund C. Lau

**Affiliations:** 1 Health Sciences Practice, Exponent, Inc., 149 Commonwealth Drive, Menlo Park, CA 94025 USA; 2 Statistical and Data Sciences Practice, Exponent, Inc., 149 Commonwealth Drive, Menlo Park, CA 94025 USA

**Keywords:** Cancer, Ecuador, Epidemiology, Leukemia, Mortality, Petroleum

## Abstract

**Purpose:**

Controversy persists over whether cancer risk is increased in communities surrounding oil fields, especially in the Oriente region of Ecuador. This ecologic study uses quantitative exposure data, updated mortality data, and improved statistical methods to study the impact of oil exploration and production activities on cancer mortality rates in the Oriente.

**Methods:**

Cancer mortality rates in the Oriente in 1990 through 2010 were compared between seven cantons with active oil exploration and production as of 1990 and thirteen cantons with little or no such activities. Poisson regression was used to estimate mortality rate ratios (RRs) adjusted for age and sex. In a two-stage analysis, canton-specific log-RRs were regressed against quantitative estimates of cumulative barrels of oil produced and well-years per canton, adjusting for canton-level demographic and socioeconomic factors.

**Results:**

Overall and site-specific cancer mortality rates were comparable between oil-producing and non-oil-producing cantons. For overall cancer mortality in males and females combined, the RR comparing oil-producing to non-oil-producing cantons was 0.85 [95 % confidence interval (CI) 0.72–1.00]. For leukemia mortality, the corresponding RR was 0.80 (95 % CI 0.57–1.13). Results also revealed no excess of mortality from acute non-lymphocytic, myeloid, or childhood leukemia. Standardized mortality ratios were consistent with RRs. Canton-specific RRs showed no pattern in relation to oil production volume or well-years.

**Conclusions:**

Results from this first ecologic study to incorporate quantitative measures of oil exploration and production showed no association between the extent of these activities and cancer mortality, including from cancers associated with benzene exposure.

**Electronic supplementary material:**

The online version of this article (doi:10.1007/s10552-013-0308-8) contains supplementary material, which is available to authorized users.

## Introduction

Little is known about the potential adverse human health impact of oil exploration and production on surrounding communities. In 1989, the International Agency for Research on Cancer (IARC) [1] determined that crude oil is “not classifiable as to its carcinogenicity in humans,” based on “inadequate evidence” for carcinogenicity in humans and “limited evidence” for carcinogenicity in experimental animals. However, questions persist about the health impact of oil exploration and production on surrounding communities. One reason for the paucity of knowledge about the potential environmental health effects of oil production is the difficulty of studying this issue rigorously. Any community health impact of oil production is not readily disentangled from the potential effects of socioeconomic status, sanitation, nutrition, health care access, lifestyle, and other health-related factors that may differ between areas with and without oil fields. Furthermore, many regions with oil fields lack high-quality, population-based data on disease incidence and/or mortality, as well as relevant data on exposure to crude oil or oil-related activities.

To date, the few studies of cancer incidence or mortality in communities with oil exploration and production activities have been ecologic in design and most have been based in the Amazon region of Ecuador, where oil extraction has taken place since 1972. Hurtig and San Sebastián [2] reported excesses in the incidence of overall and several site-specific cancers in four oil-producing cantons, compared with eleven non-oil-producing cantons, in this region in 1985–1998. Incident leukemias, but not other cancers, were also reported to be significantly more common among children in oil-producing cantons [3]. However, in an alternative analysis using cancer mortality data from the same region, Kelsh et al. [4] found no evidence that death from these cancers, or cancer overall, was higher in long-term oil-producing than non-oil-producing cantons. Combined with concerns about data quality and availability, exposure assessment, case ascertainment, population estimation, interpretation of results, and study reproducibility [5, 6], the inconsistent cancer incidence and mortality results have failed to resolve the question of whether oil production activities increase the risk of cancer in local populations.

To date, no epidemiologic studies of cancer in communities surrounding oil exploration and production activities have used quantitative information on oil-related activities. Rather, previous studies have broadly classified geographic regions as either active or not active in oil exploration and production, thereby ignoring any variation in the level of activity. To enhance prior findings by capturing the extent of oil-related activities more precisely, we sought to incorporate canton-level data on oil well locations and oil production volumes. In addition, we extended prior studies by using a more flexible and detailed statistical approach, additional years of mortality and population data, and supplemental population data on socioeconomic status, ethnicity, health care access, and residential mobility, to more thoroughly examine cancer mortality in regions with different levels of oil exploration and production activity in the Ecuadorian Amazon region.

## Methods

### Population data

Most oil exploration and production activity in Ecuador is found in the Oriente (East) region within Napo, Pastaza, Orellana, and Sucumbíos Provinces. The population and mortality data of these four provinces from 1990 through 2010 are analyzed in this study.

Population counts for cantons in the Oriente provinces in 1990, 2001, and 2010 were obtained from the Ecuador National Census (www.inec.gov.ec) [7, 8]. We also used the 2001 and 2010 census data on residential locations 5 years previously to estimate population counts in 1996 and 2005. To estimate intercensal population counts, we interpolated between the population counts in 1990, 1996, 2001, 2005, and 2010 by using a Poisson regression model that included 5-year age group, sex, year, and age–sex, age–year, and sex–year interactions to account for age- and sex-specific trends in population growth. The expected population, *P*_*ij*_, in the *i*th age group and *j*th sex group in each canton was estimated by the following Poisson model:

$$ E\left( {P_{ij} } \right) = {\text{Age}}_{i} \times {\text{Sex}}_{j} \times {\text{Year}} \times \left( {{\text{Age}}_{i} {\text{Sex}}_{j} } \right) \times \left( {{\text{Age}}_{i} {\text{Year}}} \right) \times \left( {{\text{Sex}}_{j} {\text{Year}}} \right) $$

Because Ecuador’s administrative divisions have changed in the past 30 years, statistical adjustments to the census and mortality data were made to conform to the administrative divisions in 2010 (Supplementary Table 1).

### Mortality data

Annual mortality data from 1990 through 2010 were obtained from the Ecuador National Census. We examined all cancer-related mortality and 25 site-specific cancer causes of death (Supplementary Table 2), including leukemia, childhood leukemia (ages < 15 years at diagnosis), acute non-lymphocytic leukemia (ANLL), and acute myeloid leukemia (AML, which comprised 83 % of ANLL). Death rates were analyzed based on the canton of residence at the time of death. Foreign residents who died in Ecuador were excluded. Records with missing age (0.25 %) or without a valid code for province/canton (0.06 %) were also excluded.

### Oil well and oil production data

**Fig. 1:**
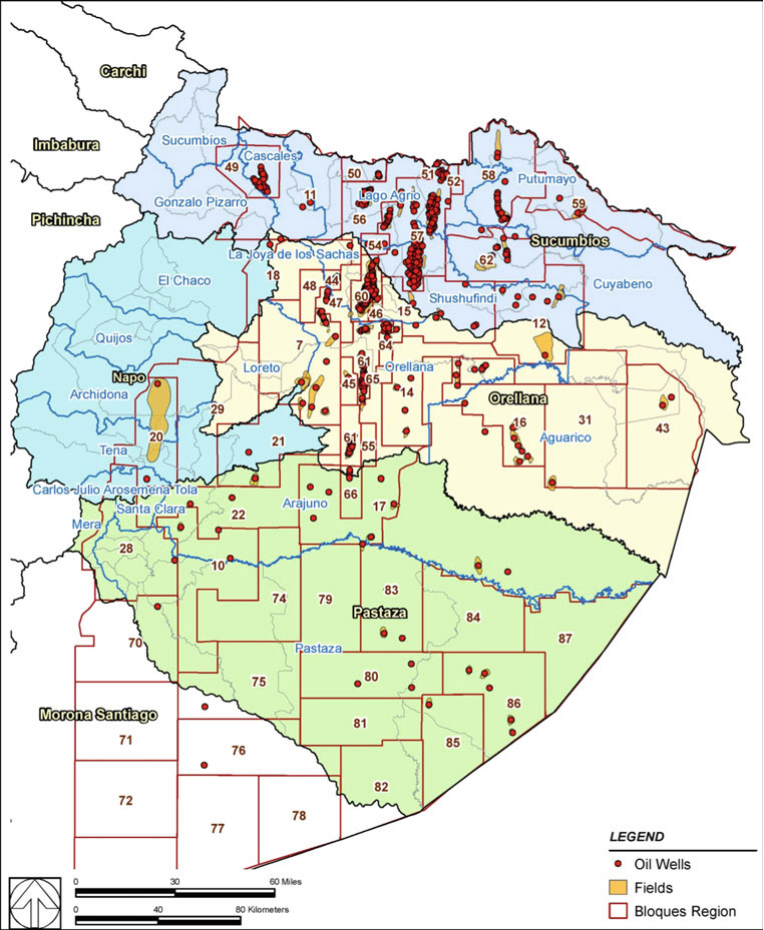
Ecuador’s northern Amazon provinces, showing oil wells, oil fields, and “bloque” areas overlaid on province and canton boundaries

To quantify the association between mortality and canton-level oil exploration and production activities, we obtained information on oil wells and oil fields from Empresa Pública Petroecuador (www.eppetroecuador.ec/idc/groups/public/documents/archivo/001373.pdf and www.eppetroecuador.ec/idc/groups/public/documents/archivo/001375.pdf). The locations of these wells and fields were overlaid on the province–canton boundaries to quantify oil exploration and production in each canton (Fig. 1). We calculated “well-year” as a measure of the cumulative number of oil wells and total years of existence within each canton (Table 1). For a more direct quantification of oil production activity in a given canton, we compiled the total volume of oil produced from 1972 to 2011 based on Petroecuador’s annual reports of oil production [9, 10] .

**Table 1:** Cumulative number of barrels of oil produced (in thousands), wells, and well-years by canton in the northern Amazon provinces of Ecuador, 1990, 2000, and 2010

Province and Canton	1990			2000			2010			Oil-Related Activity as of 1990
	Oil Production	Wells	Well-Years	Oil Production	Wells	Well-Years	Oil Production	Wells	Well-Years	
Napo										
Tena	0	2	22	641	7	56	31,357	62	417	Inactive
Archidona	0	1	11	0	1	21	0	2	32	Inactive
El Chaco	0	1	19	0	1	29	0	1	39	Inactive
Quijos	0	0	0	0	0	0	0	0	0	Inactive
Carlos Julio Arosemena Tola	0	1	18	0	1	28	0	1	38	Inactive
Napo Total	0	5	70	641	10	134	31,357	66	526	
Pastaza										
Pastaza	0	21	216	8,049	22	436	28,837	22	656	Inactive
Mera	0	0	0	0	0	0	0	0	0	Inactive
Santa Clara	0	0	0	0	0	0	0	0	0	Inactive
Arajuno	422	17	264	8,562	33	485	113,103	54	952	Inactive
Pastaza total	422	38	480	16,611	55	921	141,940	76	1,608	
Sucumbíos										
Lago Agrio	259,180	151	1,494	500,588	207	3,376	649,561	276	5,719	Active
Gonzalo Pizarro	0	0	0	0	0	0	0	0	0	Inactive
Putumayo	18,725	37	273	70,817	59	763	140,856	103	1,538	Active
Shushufindi	644,747	106	1,601	1,038,094	175	3,107	1,307,258	298	5,329	Active
Sucumbíos	0	0	0	0	0	0	0	0	0	Inactive
Cascales	9,846	36	341	26,802	51	795	50,754	75	1,469	Active
Cuyabeno	20,180	21	301	70,180	81	676	232,904	301	2,844	Active
Sucumbíos Total	952,678	351	4,010	1,706,481	573	8,717	2,381,333	1,053	16,899	
Orellana										
Francisco de Orellana	141,668	100	1,051	488,381	264	3,165	997,553	603	7,478	Active
Aguarico	0	12	129	102,281	74	533	363,587	200	2,002	Inactive
Joya de los Sachas	405,845	124	1,866	622,977	181	3,427	1,039,290	334	5,918	Active
Loreto	0	0	0	0	2	5	221	7	36	Inactive
Orellana Total	547,513	236	3,046	1,213,640	521	7,130	2,400,651	1,144	15,434	

Oil production volume was reported at the level of the oil field or “bloque” (i.e., geographic area designated for oil exploration and production). When an oil field or bloque crossed over canton boundaries, production data were divided proportionally based on the number of wells within each canton. The cumulative number of well-years and the total amount of oil produced from 1972 to 1990 were used to quantify oil exploration and production in each canton. Alternative cutoff dates were evaluated in sensitivity analyses. Seven cantons in the four provinces had a history of oil exploration and production activities as of 1990, whereas thirteen cantons had little or no oil-related activity (Table 1).

### Statistical analysis

Comparisons of overall and site-specific cancer mortality between the seven oil-producing cantons and the thirteen non-oil-producing cantons were conducted using both Poisson regression and indirect standardization. Following the age–cohort method proposed by Breslow and Day [11, 12], we used Poisson regression to model overall and site-specific cancer mortality rates as a function of age, sex, and canton-level oil exploration and production activities. The expected number of deaths, *D*_*ijk*_, was calculated from the multiplicative contributions of the *i*th age group (with ages < 35 years combined for some analyses), the *j*th sex group, the activity level of the *k*th canton, and the age-, sex-, and canton-specific person-years, *PY*_*ijk*_, and was estimated by the following Poisson model:

$$ E\left( {D_{ijk} } \right) = {\text{Age}}_{i} \times {\text{Sex}}_{j} \times {\text{Oil Activity}}_{k} \times PY_{ijk} . $$

The Oil Activity_*k*_ factor was equal to 1 if the *k*th canton was active in oil exploration and production, and 0 otherwise. The parameter associated with this factor provided an estimate of the mortality rate ratio (RR) comparing oil-producing with non-oil-producing cantons.

For comparability to prior publications that reported standardized incidence and mortality ratios [2–4], we used the indirect standardization method to estimate standardized mortality ratios (SMRs) comparing the observed with the expected number of deaths in the seven oil-producing cantons. The expected number of deaths was calculated using age- and sex-specific mortality rates from the thirteen non-oil-producing cantons and applying those rates to the person-years from the seven oil-producing cantons. For SMR analyses including males and females, the expected number of deaths was calculated as follows:

$$ {\text{Expected}} = \mathop \sum \limits_{ij} R_{ij} \times PY_{ij} $$

where *R*_*ij*_ was the mortality rate for the *i*th five-year age group and *j*th sex group in non-oil-producing cantons, and *PY*_*ij*_ was the corresponding age- and sex-specific person-years in oil-producing cantons. In SMR analyses of males and females considered separately, the expected number of deaths was summed over age-specific mortality rates and the corresponding age-specific person-years for each sex. We used the method suggested by Rothman and Boice [13] to estimate confidence intervals (CIs) and associated *p* values for the SMRs.

To further understand the variation in cancer mortality rates among the study cantons, the Poisson model was used to estimate cancer-specific mortality RRs for each of the 20 cantons, without designating particular cantons as active or inactive in oil exploration and production. We used Lago Agrio Canton in Sucumbíos Province as the reference because it had the largest population in the study area; use of a different reference group would not affect the overall results. Scatterplots were created to examine the patterns of association between the RR estimates and oil production metrics, with a nonparametric Loess regression line added to facilitate detection of any trends. To estimate the strength of association more quantitatively, we treated the Poisson regression as the first stage in the regression analysis and, as a second stage, regressed the canton-specific Poisson log-RRs as the dependent variable against canton-level oil production volume, well-years, and census-derived data on the proportion of adults who had completed high school, indigenous fraction in the population, availability of health care facilities per capita, and residential mobility in the previous 5 years. Although oil exploration and production began in the 1970s in many areas, we performed sensitivity analyses allowing for an additional 10-year induction period by relating oil production volume or well-years as of 1990 to cancer mortality in 2000–2010.

Statistical analyses were performed with SAS v9.3.

## Results

**Fig. 2:**
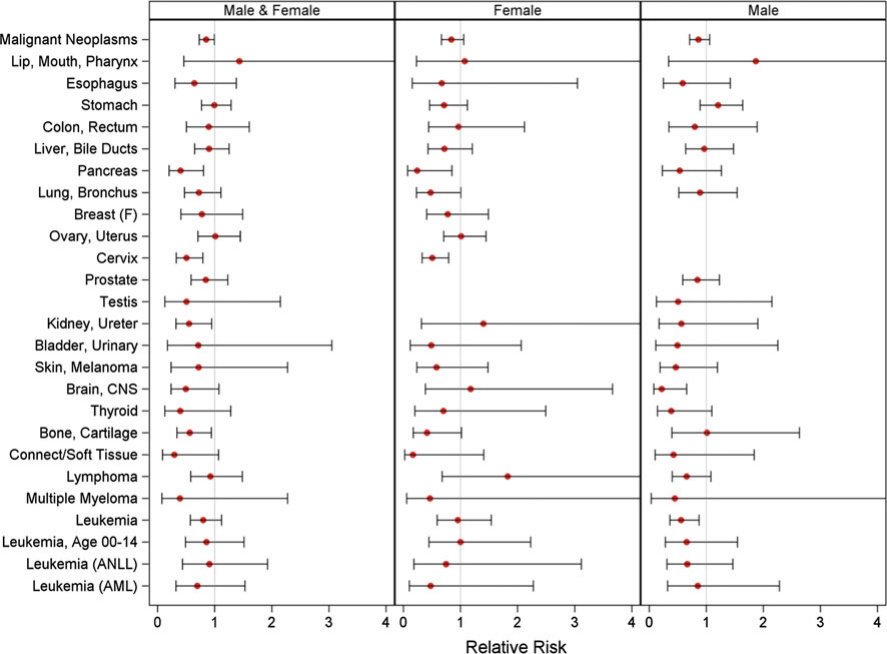
Relative risks (RRs) for all cancer and cancer-specific death comparing seven oil-producing with 13 non-oil-producing cantons, 1990–2010

Demographic characteristics and cancer mortality rates of populations residing in the four northern Amazon provinces are summarized in Supplementary Tables 3 and 4. Results from the Poisson regression analysis of cancer mortality in oil-producing versus non-oil-producing cantons among males and females analyzed together and separately are shown in Fig. 2. The corresponding numerical results from both the Poisson regression and SMR analyses are shown in Table 2. For males and females combined, the RR for all cancer-related deaths was 0.85 (95% CI 0.72–1.00) comparing the seven oil-producing cantons with the thirteen non-oil-producing cantons. When males and females were analyzed separately, the RRs showed a similar deficit. We found few consistent elevations in the mortality rate of any site-specific cancer in oil-producing versus non-oil-producing cantons based on either RRs or SMRs in males and females together or separately. Ten or fewer deaths were identified in the oil-producing cantons for each of the following cancers, resulting in imprecise RR estimates: lip/mouth/pharynx (the only cancer for which RR estimates were >1.0 in males, females, and both sexes combined), testis, skin, thyroid, kidney, bladder, and multiple myeloma.

**Table 2:** Observed and expected cancer deaths and standardized mortality ratios (SMR) and Poisson rate ratios for cancer mortality in cantons active or inactive in oil exploration and production activities in the northern Amazon provinces of Ecuador, 1990–2010

Cause of Cancer Death	Sex	Deaths (Active)	Crude rate (Active)	Deaths (Inactive)	Crude rate (Inactive)	Expected deaths	SMR		Rate ratio	
							95 % CI	*p* value	95 % CI	*p* value
All malignant neoplasms	M + F	682	17.51	872	25.13	878.0	0.78 (0.72–0.84)	<.001	0.85 (0.72–1.00)	0.048
	M	379	17.76	438	24.46	467.8	0.81 (0.73–0.90)	<.001	0.86 (0.70–1.06)	0.165
	F	303	17.20	434	25.83	410.3	0.74 (0.66–0.83)	<.001	0.84 (0.66–1.06)	0.145
Lip, mouth, pharynx	M + F	8	0.21	6	0.17	5.9	1.35 (0.58–2.66)	0.488	1.43 (0.45–4.51)	0.541
	M	5	0.23	3	0.17	3.1	1.60 (0.52–3.73)	0.412	1.87 (0.33–10.43)	0.475
	F	3	0.17	3	0.18	2.8	1.08 (0.22–3.14)	1.000	1.07 (0.22–5.19)	0.928
Esophagus	M + F	11	0.28	17	0.49	16.9	0.65 (0.32–1.16)	0.174	0.65 (0.30–1.38)	0.259
	M	8	0.37	13	0.73	13.4	0.60 (0.26–1.18)	0.164	0.59 (0.24–1.43)	0.237
	F	3	0.17	4	0.24	3.5	0.85 (0.17–2.49)	1.000	0.67 (0.15–3.05)	0.603
Stomach	M + F	153	3.93	161	4.64	158.4	0.97 (0.82–1.13)	0.703	0.99 (0.76–1.29)	0.959
	M	108	5.06	86	4.80	90.5	1.19 (0.98–1.44)	0.079	1.21 (0.89–1.64)	0.233
	F	45	2.55	75	4.46	68.0	0.66 (0.48–0.89)	0.004	0.71 (0.45–1.12)	0.145
Colon, rectum	M + F	22	0.56	24	0.69	23.4	0.94 (0.59–1.42)	0.873	0.90 (0.50–1.61)	0.717
	M	10	0.47	11	0.61	11.3	0.89 (0.43–1.63)	0.859	0.80 (0.34–1.90)	0.609
	F	12	0.68	13	0.77	12.2	0.99 (0.51–1.72)	1.000	0.96 (0.44–2.13)	0.926
Liver, bile ducts	M + F	65	1.67	70	2.02	70.0	0.93 (0.72–1.18)	0.597	0.90 (0.64–1.26)	0.538
	M	41	1.92	35	1.95	37.0	1.11 (0.80–1.50)	0.550	0.97 (0.63–1.48)	0.876
	F	24	1.36	35	2.08	33.1	0.73 (0.47–1.08)	0.125	0.72 (0.42–1.21)	0.212
Pancreas	M + F	11	0.28	27	0.78	27.3	0.40 (0.20–0.72)	<.001	0.40 (0.20–0.81)	0.011
	M	8	0.37	14	0.78	15.0	0.53 (0.23–1.05)	0.074	0.53 (0.23–1.27)	0.154
	F	3	0.17	13	0.77	12.3	0.24 (0.05–0.71)	0.004	0.24 (0.07–0.86)	0.028
Lung, bronchus	M + F	36	0.92	49	1.41	48.8	0.74 (0.52–1.02)	0.069	0.72 (0.47–1.11)	0.139
	M	26	1.22	27	1.51	28.9	0.90 (0.59–1.32)	0.671	0.89 (0.51–1.55)	0.676
	F	10	0.57	22	1.31	19.9	0.50 (0.24–0.93)	0.023	0.48 (0.23–1.01)	0.053
Breast (female)	F	16	0.91	21	1.25	20.5	0.78 (0.45–1.27)	0.376	0.78 (0.40–1.49)	0.444
Ovary, uterus	F	59	3.35	60	3.57	57.2	1.03 (0.79–1.33)	0.842	1.01 (0.70–1.45)	0.957
Cervix	F	28	1.59	56	3.33	54.8	0.51 (0.34–0.74)	<.001	0.51 (0.32–0.80)	0.004
Prostate	M	50	2.34	60	3.35	58.4	0.86 (0.64–1.13)	0.299	0.85 (0.58–1.24)	0.389
Testis	M	3	0.14	5	0.28	5.6	0.54 (0.11–1.56)	0.378	0.51 (0.12–2.16)	0.357
Kidney, ureter	M + F	8	0.21	9	0.26	9.9	0.81 (0.35–1.60)	0.691	0.55 (0.32–0.95)	0.034
	M	4	0.19	6	0.34	7.1	0.57 (0.15–1.45)	0.332	0.56 (0.17–1.91)	0.354
	F	4	0.23	3	0.18	2.8	1.42 (0.38–3.64)	0.620	1.40 (0.31–6.34)	0.658
Bladder, other urinary organs	M + F	4	0.10	6	0.17	6.3	0.63 (0.17–1.62)	0.482	0.71 (0.17–3.06)	0.649
	M	3	0.14	6	0.34	6.3	0.47 (0.10–1.38)	0.245	0.50 (0.11–2.26)	0.362
	F	1	0.06	0	0.00	0.0				
Skin, melanoma	M + F	5	0.13	7	0.20	7.3	0.69 (0.22–1.60)	0.533	0.72 (0.23–2.28)	0.576
	M	3	0.14	5	0.28	5.2	0.58 (0.12–1.69)	0.478	0.47 (0.18–1.20)	0.114
	F	2	0.11	2	0.12	2.1	0.96 (0.11–3.46)	1.000	0.58 (0.23–1.49)	0.257
Brain, central nervous system	M + F	12	0.31	24	0.69	25.5	0.47 (0.24–0.82)	0.005	0.50 (0.23–1.08)	0.078
	M	4	0.19	16	0.89	17.8	0.22 (0.06–0.57)	<.001	0.22 (0.07–0.66)	0.007
	F	8	0.45	8	0.48	7.7	1.04 (0.45–2.05)	1.000	1.18 (0.38–3.66)	0.777
Thyroid	M + F	5	0.13	9	0.26	8.7	0.58 (0.18.5–1.34)	0.271	0.40 (0.12–1.29)	0.123
	M	1	0.05	3	0.17	3.0	0.33 (0.004–1.85)	0.395	0.39 (0.14–1.11)	0.076
	F	4	0.23	6	0.36	5.7	0.70 (0.19–1.80)	0.658	0.70 (0.20–2.50)	0.585
Bone, articular cartilage	M + F	12	0.31	16	0.46	16.1	0.75 (0.39–1.30)	0.376	0.56 (0.33–0.95)	0.032
	M	9	0.42	8	0.45	8.5	1.05 (0.48–2.00)	0.963	1.01 (0.39–2.64)	0.976
	F	3	0.17	8	0.48	7.5	0.40 (0.08–1.16)	0.114	0.41 (0.17–1.03)	0.057
Connective/soft tissue	M + F	3	0.08	10	0.29	9.9	0.30 (0.06–0.88)	0.022	0.29 (0.08–1.08)	0.065
	M	2	0.09	4	0.22	4.1	0.48 (0.05–1.75)	0.436	0.43 (0.10–1.85)	0.252
	F	1	0.06	6	0.36	5.8	0.17 (0.002–0.96)	0.041	0.17 (0.02–1.42)	0.100
Lymphoma	M + F	35	0.90	34	0.98	37.6	0.93 (0.65–1.30)	0.751	0.92 (0.58–1.49)	0.746
	M	24	1.12	28	1.56	31.7	0.76 (0.48–1.13)	0.191	0.66 (0.40–1.09)	0.102
	F	11	0.62	6	0.36	5.9	1.88 (0.94–3.36)	0.074	1.83 (0.67–4.98)	0.237
Multiple myeloma	M + F	2	0.05	3	0.09	2.8	0.72 (0.08–2.59)	0.947	0.39 (0.07–2.28)	0.296
	M	1	0.05	1	0.06	0.9	1.09 (0.01–6.09)	1.000	0.45 (0.03–7.34)	0.573
	F	1	0.06	2	0.12	1.9	0.53 (0.007–2.97)	0.885	0.47 (0.05–4.43)	0.504
Leukemia	M + F	64	1.64	73	2.10	80.6	0.79 (0.61–1.01)	0.066	0.80 (0.57–1.13)	0.199
	M	34	1.59	47	2.62	54.7	0.62 (0.43–0.87)	0.004	0.56 (0.36–0.88)	0.012
	F	30	1.70	26	1.55	25.9	1.16 (0.78–1.66)	0.464	0.95 (0.59–1.55)	0.850
Leukemia, childhood (00–14 years)	M + F	23	1.46	25	1.70	26.6	0.86 (0.55–1.30)	0.559	0.85 (0.48–1.52)	0.585
	M	10	1.16	12	1.57	13.6	0.74 (0.35–1.35)	0.409	0.65 (0.28–1.55)	0.327
	F	13	1.83	13	1.83	13.0	1.00 (0.53–1.71)	1.000	1.00 (0.44–2.24)	0.995
Leukemia, acute non–lymphocytic	M + F	15	0.39	15	0.43	16.9	0.89 (0.50–1.47)	0.766	0.91 (0.43–1.93)	0.807
	M	10	0.47	9	0.50	10.7	0.93 (0.45–1.71)	0.981	0.67 (0.30–1.47)	0.316
	F	5	0.28	6	0.36	6.1	0.82 (0.26–1.91)	0.852	0.75 (0.18–3.12)	0.688
Leukemia, acute myeloid	M + F	11	0.28	14	0.40	15.6	0.71 (0.35–1.26)	0.298	0.70 (0.32–1.53)	0.370
	M	8	0.37	8	0.45	9.5	0.85 (0.36–1.67)	0.795	0.85 (0.32–2.28)	0.747
	F	3	0.17	6	0.36	6.1	0.49 (0.10–1.43)	0.279	0.48 (0.10–2.28)	0.351

Rates are per 100,000 person-years

F: female; M: male

Mortality from leukemia was not elevated in oil-producing compared with non-oil-producing cantons (Fig. 2). Likewise, mortality from ANLL or AML was not higher in cantons that were active in oil exploration and production, although results were based on small numbers of deaths. Leukemia-related mortality among children up to age 14 years also was not associated with the presence of oil-related activities. Of all the specific cancer sites examined, only mortality from cancer of the lip, mouth, and pharynx was elevated among both males and females in the oil-producing cantons, but estimates were statistically unstable. Classification of canton-level oil production status according to the system used by [2] yielded no substantial difference (data not shown).

**Fig. 3:**
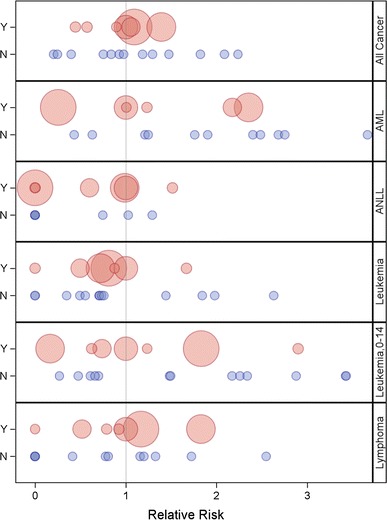
Relative risk of all cancer and leukemia-related death in the seven cantons with oil exploration and production activity (shown in *red*; “Y” = yes, oil-producing) and thirteen cantons with little or no oil exploration and production activity (shown in *blue*; “N” = no, not oil-producing) in northern Amazon provinces in Ecuador, 1990–2010. The size of each *red circle* is proportional to the cumulative volume of oil produced as of 1990. AML = acute myeloid leukemia; ANLL = acute non-lymphocytic leukemia; leukemia, 0–14 = leukemia in children aged 0–14 years. (Color figure online)

**Fig. 4:**
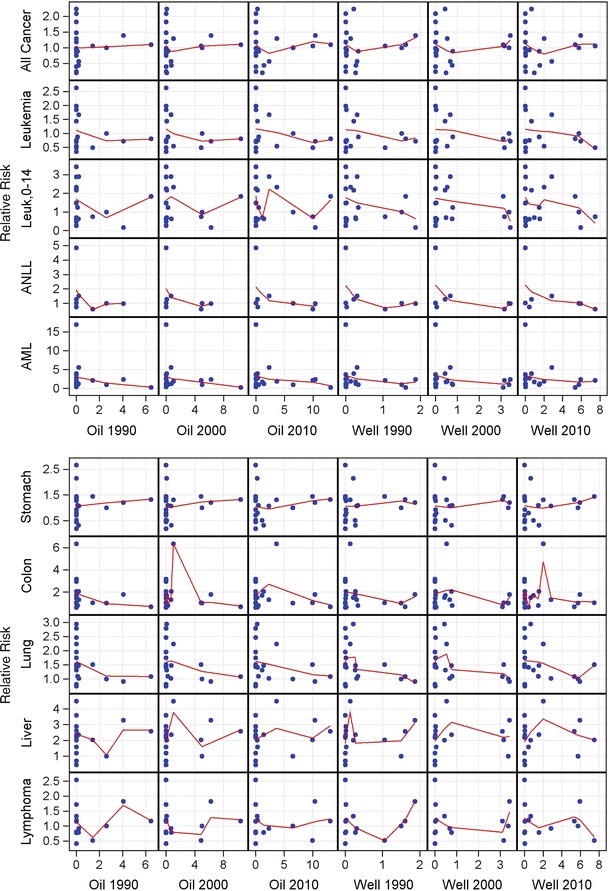
Scatterplots of relative risk for overall and site-specific cancer mortality versus cumulative well-years (per 1,000) and oil produced (per 100 million barrels) as of 1990, 2000, and 2010, with nonparametric Loess regression lines. AML = acute myeloid leukemia; ANLL = acute non-lymphocytic leukemia; leuk, 0–14 = leukemia in children aged 0–14 years

The 20 canton-specific RRs from the Poisson regression analysis of overall and site-specific cancer mortality (with Lago Agrio as the reference, RR = 1), adjusted for age and sex, are shown in Fig. 3. The figure reveals no apparent association between oil-related activity in each canton and the RRs for mortality from overall cancer, overall leukemia, childhood leukemia, ANLL, AML, or lymphoma. Rank-ordering of the RRs showed no apparent patterns to suggest increased RRs in the oil-producing cantons. Among the oil-producing cantons, the magnitude of the RR bore no relationship with the amount of oil produced, as represented by the size of the markers in Fig. 3. Due to sparse numbers for AML and ANLL, RRs could not be estimated in some cantons with insufficient data. Scatterplots of the RRs for mortality from overall cancer and other major cancer sites against total volume of oil produced or total well-years also showed no consistent differences in cancer mortality according to level of oil production activity (Fig. 4).

To further examine the association between cancer mortality and the extent of oil exploration and production activities, the age- and sex-adjusted canton-specific log-RRs were regressed against canton-level barrels of oil produced (per 100 million) and well-years (per 1,000) as of 1990, 2000, and 2010, with or without adjustment for educational attainment, indigenous fraction, and health care facilities per capita. Residential mobility was not associated with overall or site-specific cancer mortality and therefore was not included. After multivariate adjustment, no consistent, stable positive associations were observed between the two metrics of oil production and exploration activities and site-specific cancer mortality rates (Table 3). When the analysis was not adjusted for canton-level educational attainment, indigenous fraction, or health care facilities (data not shown), most of the results were not meaningfully different. Well-years but not oil production was positively associated with mortality from cancer of the lip, mouth, and pharynx, whereas inverse associations were detected with mortality from multiple myeloma and cancers of the pancreas, testis, thyroid, and bladder and other urinary organs. Sensitivity analyses allowing for a 10-year induction period revealed no consistent positive associations with overall or site-specific cancer mortality (data not shown).

**Table 3:** Associations of age- and sex-adjusted canton-specific log-relative risks of cancer mortality with barrels of oil produced or well-years in 1990, adjusted for canton-level educational attainment, percent indigenous, and health care facilities per capita, northern Amazon provinces of Ecuador, 1990–2010

Cause of Death	Oil Production (per 100 million barrels)		Well-Years (per 1,000)	
	Estimate (95 % CI)	*p* value	Estimate (95 % CI)	*p* value
All malignant neoplasms	1.04 (0.74, 1.45)	0.807	1.07 (0.40, 2.86)	0.889
Lip, mouth, pharynx	0.94 (0.56, 1.57)	0.788	4.09 (0.90, 18.60)	0.066
Esophagus	0.82 (0.48, 1.40)	0.446	2.54 (0.52, 12.28)	0.226
Stomach	0.99 (0.66, 1.46)	0.943	1.22 (0.38, 3.90)	0.721
Colon, rectum	0.87 (0.57, 1.31)	0.475	1.35 (0.40, 4.58)	0.602
Liver, bile ducts	1.10 (0.69, 1.75)	0.654	1.08 (0.28, 4.23)	0.903
Pancreas	1.15 (0.83, 1.60)	0.383	0.35 (0.13, 0.93)	0.038
Lung, bronchus	0.96 (0.75, 1.23)	0.735	0.93 (0.46, 1.91)	0.841
Breast (female)	1.08 (0.71, 1.66)	0.698	0.74 (0.21, 2.60)	0.616
Ovary, uterus	0.87 (0.56, 1.35)	0.518	1.84 (0.51, 6.65)	0.328
Cervix	0.93 (0.73, 1.18)	0.506	1.33 (0.59, 2.96)	0.433
Prostate	0.98 (0.65, 1.47)	0.904	1.13 (0.34, 3.74)	0.831
Testis	0.75 (0.42, 1.37)	0.325	0.87 (0.15, 4.98)	0.864
Kidney, ureter	0.93 (0.40, 2.20)	0.845	0.84 (0.03, 26.63)	0.904
Bladder, other urinary organs	0.94 (0.61, 1.46)	0.776	0.52 (0.14, 1.90)	0.299
Skin, melanoma	1.38 (0.01, 351.13)	0.827	3.51 (0.00, –.–)	0.824
Brain, central nervous system	0.92 (0.48, 1.77)	0.759	1.23 (0.11, 13.39)	0.836
Thyroid	0.95 (0.62, 1.45)	0.806	0.47 (0.13, 1.61)	0.209
Bone, articular cartilage	1.06 (0.72, 1.56)	0.736	0.53 (0.17, 1.66)	0.255
Connective/soft tissue	–.–		–.–	
Lymphoma	1.12 (0.75, 1.67)	0.547	0.79 (0.21, 2.93)	0.690
Multiple myeloma	1.01 (0.63, 1.61)	0.968	0.37 (0.09, 1.45)	0.140
Leukemia	1.01 (0.77, 1.33)	0.917	0.71 (0.30, 1.63)	0.381
Leukemia, childhood (00–14 y)	1.24 (0.77, 1.98)	0.352	0.32 (0.08, 1.29)	0.102
Leukemia, acute non-lymphocytic	1.54 (0.20, 12.06)	0.459	0.26 (0.00, 25.75)	0.335
Leukemia, acute myeloid	0.60 (0.35, 1.04)	0.066	2.14 (0.43, 10.60)	0.323

Educational attainment: proportion of canton residents aged 25 years and older educated at the high school level or above

CI: confidence interval

## Discussion

In this ecologic study, we found no evidence of increased overall or site-specific cancer mortality in association with increased level of oil exploration and production activities in the Oriente region of Ecuador. Whether oil-related activity was classified broadly or more finely based on well-years or volume of oil produced, and whether using the traditional SMR approach or the more flexible and detailed Poisson regression approach, we observed no apparent excess of cancer mortality in cantons with more oil exploration and production.

On the contrary, for several cancer sites, mortality was markedly lower in oil-producing than non-oil-producing cantons. If oil-producing cantons have more complete and accurate reporting of cause of death than non-oil-producing cantons due to greater access to mainstream health services as a result of oil-related economic activity, then differential outcome classification would be likely to result in overestimated—not underestimated—RRs. Given that the proportion of death certificates signed by a physician was similar between oil-producing (65 %) and non-oil-producing (58 %) cantons, and census measures of access to health care were also comparable between regions, it is improbable that information bias due to poorer vital statistics reporting in oil-producing than non-oil-producing cantons accounts for the absence of an observed association with cancer mortality. Instead, another plausible explanation for the observed deficits of cause-specific mortality in oil-producing cantons may be unmeasured differences in behavioral, social, cultural, and/or structural factors, rather than a direct beneficial effect of oil-related activities. This explanation is consistent with the fact that most associations were weaker in magnitude after adjustment for canton-level education attainment, indigenous fraction, and density of health care facilities.

Information potentially relevant to the evaluation of the health effects of crude oil exposure can be derived from occupational health studies of oil exploration and production workers. To our knowledge, five cohort studies (each with multiple publications) [14–19] and five case–control studies [6, 20–23] have evaluated cause-specific mortality and/or cancer incidence among oil exploration and production (i.e., “upstream”) workers. Overall, no clear picture of excess risk of cancer incidence or mortality has emerged from these studies, and no cancers occurred in significant excess in the majority of studies.

Kelsh et al. [4] found that liver cancer mortality in the Ecuadorian Amazon region in 1990–2005 was elevated in cantons with oil production activities. None of the occupational studies described above detected an excess of liver cancer incidence or mortality among upstream petroleum industry workers, nor did we detect such an excess in our updated analysis. Hepatitis B virus (HBV), the cause of the majority of liver cancer worldwide [24], is endemic in the Amazon basin, where 2–14 % of the population is chronically infected, with differences in the prevalence of chronic infection by ethnicity and geographic region [25, 26]. Thus, chronic HBV infection may be a major determinant of regional differences in liver cancer incidence and mortality in the Amazon region of Ecuador.

In the study by Hurtig and San Sebastián [2], the two malignancies that accounted for the greatest proportion of the excess overall cancer risk were stomach cancer in men and cervical cancer in women. Neither of these malignancies was consistently positively associated with oil-related activities in our study, the study by Kelsh et al. [4], or the occupational studies of upstream petroleum industry workers described above. The primary causes of these cancers are also infectious agents, namely *Helicobacter pylori* as the leading cause of stomach cancer (and a minority of lymphomas), and oncogenic human papillomaviruses (HPV) as the leading cause of cervical cancer (and a substantial proportion of anogenital and oropharyngeal cancers) [24]. Both of these infections are common worldwide, including in Ecuador [27, 28]. The prevalence of *H. pylori* infection varies by individual- and area-level socioeconomic status, urbanization, sanitation, water quality, health care access, ethnicity, and birthplace [29, 30], while the prevalence of HPV infection varies by sexual behavior, which in turn depends on population migration and social, cultural, religious, and economic factors [31]. Any of these determinants could explain the observed geographic differences in stomach and cervical cancer incidence in the Ecuadorian Amazon region. Furthermore, disparities in cervical cancer incidence and mortality in Latin American countries have been attributed to differential access to cervical cancer screening and treatment [32].

The other findings of Hurtig and San Sebastián [2, 3], including excesses of incident cancers of the rectum, connective/soft tissue, kidney, uterine cervix, and lymph nodes and childhood leukemia, were not confirmed by our study or most studies of oil exploration and production workers. However, Yang and Zhang [33] observed an excess of leukemia around oil fields in China, and Gazdek et al. [34] reported a significant excess of certain hematopoietic malignancies, albeit not lymphomas or all leukemias combined, in Croatian populations living near oil and natural gas fields.

A key methodological difference that may explain part of the inconsistency in results across studies is our use of cancer mortality rather than incidence data. Hurtig and San Sebastián [2, 3], Yang and Zhang [33], and Gazdek et al. [34] used cancer incidence data, which more accurately reflect the risk of developing disease than cancer mortality data, especially for cancer types with relatively high survival. However, in regions that lack mandatory population-based cancer surveillance, incident cases may be missed and those that are reported may represent a biased sample of total incident cases. For example, Hurtig and San Sebastián were able to include only incident cancer cases diagnosed in Quito and reported to the National Tumor Registry with a permanent residence in the Amazon region [2, 3, 35]. Suspected cancer cases in the Amazon region are referred to the capitol city of Quito for diagnosis and treatment, but the long distance—requiring as much as a 12-hour bus ride—and cultural differences between the Amazon and Quito most likely pose a substantial barrier to many residents of the study area. Therefore, cancer incidence among residents of the Amazon region may be grossly underreported, and cancer cases identified in the National Tumor Registry may differ considerably from unreported cases in terms of disease characteristics, patient attributes, and exposures. For example, it is conceivable that cancer cases in oil-producing areas, compared with those in non-oil-producing areas, have better access to navigable roads and/or transportation, enabling them to travel to Quito for diagnosis and treatment and resulting in overestimated relative risks.

By contrast, our study used mortality data abstracted from death certificates. Mortality rate ratios are unbiased estimates of incidence rate ratios if the exposure of interest does not affect disease survival or reporting. Currently, no evidence shows that proximity to oil exploration and production activities influences cancer survival. Compared with cancer incidence data in the Ecuadorian Amazon region, mortality data are likely to be more complete and less systematically biased. However, the use of death certificate data, especially in developing regions, entails important limitations in data quality and population coverage. First, the accuracy of the recorded cause of death depends on the diagnostic abilities of the responsible medical facility and/or physician. Second, mortality data may be deficient due to incomplete coverage of the civil registration system, leading to under-registration of deaths by an estimated 13.5 % [36] to 30 % [37], and such under-registration may be unequal between oil-producing and non-oil-producing regions. Third, all death records had a cause of death listed, but 25 % of deaths in oil-producing cantons and 28 % in non-oil-producing cantons had “symptoms and ill-defined conditions” identified as the cause of death. Thus, misclassification of causes of death was undoubtedly present, with potential variation across cantons in accordance with degree of development, access to medical care, and other demographic and socioeconomic factors, leading to an unknown impact on the results.

Other differences between our analysis and those of Hurtig and San Sebastián include the definitions of areas with or without oil exploration and production, and methods for estimating the annual population at risk in the study area. We used information on well and oil field locations, drilling dates, and oil field production volumes to characterize the extent of oil exploration and production activities. By contrast, the sources and methods used by Hurtig and San Sebastián were not clearly specified and resulted in different classifications than ours [2, 3], although our results were similar when using their classification. We used data from the 1990, 2001, and 2010 national censuses and imputed canton-, age-, and sex-specific population denominators for each intercensal year. Hurtig and San Sebastián used population projections for 1992 or 1993, based on the 1990 national census, as denominators for cancer incidence rates in 1985–1998 or 1985–2000. The latter approach almost certainly underestimated post-1990 populations. Based on national census data showing that oil-producing populations grew more quickly than non-oil-producing populations between 1990 and 2001, this approach would have resulted in overestimated relative risks.

Our study and previous investigations of cancer in communities surrounding oil fields [2–4, 33–35, 38] are all limited by their ecologic design, in which exposure status is assigned at the community level. By assuming that all individuals within a given community have the same exposure status, such studies introduce an unknown degree and direction of bias, as associations observed at the community level may not apply at the individual level. Furthermore, ecologic studies such as these have limited information on potential confounders that may explain observed differences in disease rates between populations. Most studies, including ours, have modest numbers of most site-specific cancers, with correspondingly limited statistical power and ability to control for confounding. An additional concern is that none of these studies can account fully for residential migration and therefore could not classify individuals according to the canton in which they resided for the longest duration or at a biologically relevant latency period prior to death. In fact, biologically plausible latency periods between exposure to oil-related contaminants and cancer diagnosis or death are not established. Finally, we were unable to assess the validity and completeness of oil well and oil production data or cancer mortality data. Even if exposure and outcome misclassification were random, however, the resulting bias would not necessarily attenuate the estimated RRs [39–41]. Given these substantial limitations, the reported associations cannot be interpreted as definitively establishing or refuting a causal effect of crude oil on cancer incidence or mortality.

Ideally, studies of the association between residence near oil fields and risk of cancer should use individual-level data on exposure to crude oil and its waste products, as well as abundant data on potential confounders. However, no studies of environmental exposure to oil exploration and production activities have collected such information, and unbiased prospective collection of such data is now virtually impossible, in the aftermath of immense public scrutiny and controversy concerning oil exploration and production in the Ecuadorian Amazon region, along with close involvement of local organizations in setting the agenda of research in the region [42].

Despite these caveats, our study offers several advantages over previously published studies of health outcomes in the Ecuadorian Amazon region. These strengths include more years of follow-up, allowing for longer latency periods and larger sample sizes; more detailed, quantitative information on oil exploration and production; a more refined approach to data analysis; and adjustment for potential confounding by demographic and socioeconomic factors. In particular, a key advantage of the Poisson regression method over the more conventional SMR method is the ability to accommodate a multi-category or continuous rather than binary exposure variable.

In conclusion, in this extended and enhanced analysis of cancer mortality in the Oriente region of Ecuador from 1990 through 2010, we observed no apparent excess of death from any or all cancers in areas with oil exploration and production activities, compared with areas that had little or no oil-related activity. Given the methodological limitations of this study, our findings do not necessarily indicate that exposure to crude oil and oil-related activities is causally unrelated to any form of cancer. However, our findings provide no evidence to support such a relationship and further demonstrate that in the Ecuadorian Amazon region, residing near oil fields appears not to adversely affect cancer mortality.

## Electronic supplementary material

Below is the link to the electronic supplementary material.

Supplementary material 1 (DOCX 32 kb)
