# Supplementary material for: Cancer mortality and quantitative oil production in the Amazon region of Ecuador, 1990–2010
Source: Cancer Causes Control. 2013 Nov 30;25(1):59–72. doi: 10.1007/s10552-013-0308-8 (PMC3889987; doi:10.1007/s10552-013-0308-8)
Supplement: Supplementary file 1 — Supplementary material 1 (DOCX 32 kb) [file 10552_2013_308_MOESM1_ESM.docx]

**Supplementary Table 1.** Provinces and cantons established since 1990 in the northern Amazon provinces of Ecuador^[[1]](#footnote-1)^

| New Entity | Year | Source Entities |
| --- | --- | --- |
| Loreto Canton | 1992 | Loreto, Avila, and Puerto Murialdo Parishes (Archidona Canton); San José de Payamino Parish (Quijos Canton) |
|  |  |  |
| Orellana Province | 1998 | Orellana, Loreto, Aguarico, and La Joya de los Sachas Cantons (Napo Province) |
|  |  |  |
| Carlos Julio Arosemana Tola Canton | 1998 | Carlos Julio Arosemana Tola Parish (Tena Canton, Napo Province) |
|  |  |  |
| Cuyabeno Canton | 1998 | Cuyabeno, Tarapoa, and Aguas Negras Parishes (Lago Agrio Canton, Sucumbíos Province) |
|  |  |  |
| Santa Clara Canton | 1992 | Santa Clara Parish (Pastaza Canton, Pastaza Province) |
|  |  |  |
| Arajuno Canton | 1998 | Arajuno, Curaray Parish (Pastaza Canton, Pastaza Province) |

**Supplementary Table 2.** International Classification of Diseases, 9^th^ and 10^th^ revisions (ICD-9 and ICD-10) codes for cancer-related causes of death

| Cancer Type | ICD-9 | ICD-10 |
| --- | --- | --- |
| All malignant neoplasms | 140-208 | C00-C97 |
| Lip, oral cavity and pharynx | 140-149 | C00-C14 |
| Esophagus | 150 | C15 |
| Stomach | 151 | C16 |
| Colon and rectum | 153, 159.0, 154.0-154.1 | C18-C20, C26.0 |
| Liver and intrahepatic bile ducts | 155 | C22 |
| Pancreas | 157 | C25 |
| Lung including trachea and bronchus | 162 | C33-C34 |
| Breast | 174-175 | C50 |
| Ovary and uterus adnexa | 179, 182, 183.0 | C54-C56 |
| Cervix uteri | 180 | C53 |
| Prostate | 185 | C61 |
| Testis | 186 | C62 |
| Kidney, renal pelvis, and ureter | 189.0-189.2 | C64-C66 |
| Bladder, urethra, and other urinary organs | 188, 189.3-189.4, 189.8-189.9 | C67-C68 |
| Skin and melanoma | 172-173 | C43-C44 |
| Brain and other parts of nervous system | 191-192 | C70-C72 |
| Thyroid | 193 | C73 |
| Bone and articular cartilage | 170 | C40-C41 |
| Connective and other soft tissue | 171 | C49 |
| Lymphoma | 200, 201, 202.0-202.2, 202.8-202.9 | C81-C85, C96.3 |
| Multiple myeloma | 203.0, 238.6 | C90.0, C90.2 |
| Leukemia | 204-208 | C91-C95 |
| Acute non-lymphocytic leukemia | 205.0, 206.0, 207.0 | C92.0, C92.4, C92.5, C92.6, C93.0, C94.0, C94.2, C94.4 |
| Acute myeloid leukemia | 205.0 | C92.0, C92.4-C92.6 |

**Supplementary Table 3.** Demographic characteristics of populations in the northern Amazon provinces of Ecuador, 1990 and 2010

|  | Napo | | Orellana | | Pastaza | | Sucumbíos | | Ecuador | |
| --- | --- | --- | --- | --- | --- | --- | --- | --- | --- | --- |
| Characteristic | 1990 | 2010 | 1990 | 2010 | 1990 | 2010 | 1990 | 2010 | 1990 | 2010 |
| Total population | 56,297 (100.0) | 103,697 (100.0) | 47,090 (100.0) | 136,396 (100.0) | 41,811 (100.0) | 83,933 (100.0) | 76,952 (100.0) | 176,472 (100.0) | 9,648,189 (100.0) | 14,483,499 (100.0) |
| Age (mean/SD) | 20.3 (17.2) | 24.1 (18.9) | 20.3 (16.7) | 23.5 (18.1) | 21.9 (17.7) | 24.6 (19.3) | 20.8 (16.6) | 24.9 (18.6) | 24.1 (19.0) | 28.4 (20.5) |
| Age (25^th^, 50^th^, 75^th^ pct, years) | 7, 16, 30 | 9, 19, 36 | 7, 17, 30 | 9, 20, 35 | 8, 18, 32 | 9, 20, 36 | 7, 17, 30 | 10, 21, 37 | 9, 20, 35 | 11, 25, 42 |
| Age < 10 years (%) | 18,746 (33.3) | 27,652 (26.7) | 15,498 (32.9) | 36,824 (27.0) | 12,390 (29.6) | 21,743 (25.9) | 24,214 (31.5) | 43,586 (24.7) | 2,515,085 (26.1) | 2,989,083 (20.6) |
| Age > 64 years (%) | 1,309 (2.3) | 4,096 (3.9) | 840 (1.8) | 4,236 (3.1) | 1,204 (2.9) | 3,716 (4.4) | 1,340 (1.7) | 6,553 (3.7) | 418,183 (4.3) | 940,905 (6.5) |
| Male (%) | 28,764 (51.1) | 52,774 (50.9) | 25,688 (54.6) | 72,130 (52.9) | 22,005 (52.6) | 42,260 (50.4) | 42,416 (55.1) | 92,848 (52.6) | 4,796,412 (49.7) | 7,177,683 (49.6) |
| Rural residence (%) | NR | 68,264 (65.8) | NR | 80,468 (59.0) | NR | 47,006 (56.0) | NR | 103,432 (58.6) | NR | 5,392,713 (37.2) |
| Indigenous (%) | NR | 58,845 (56.7) | NR | 43,329 (31.8) | NR | 33,399 (39.8) | NR | 23,684 (13.4) | NR | 1,018,176 (7.0) |
| Literate (%)^a^ | 14,271 (76.5) | 39,105 (91.3) | 12,964 (80.4) | 51,336 (91.2) | 12,045 (79.7) | 31,872 (90.5) | 23,253 (85.0) | 70,436 (91.1) | 3,336,443 (84.4) | 6,615,348 (91.3) |
| Completed high school (%)^a,b^ | 11,144 (19.8) | 21,241 (49.6) | 6,864 (14.6) | 23,685 (42.0) | 51,18 (33.9) | 19,237 (54.5) | 11,805 (28.2) | 33,042 (42.8) | 2,851,388 (29.6) | 3,728,561 (51.5) |
| Currently married (%)^a^ | 13,966 (74.8) | 24,419 (57.0) | 10,614 (65.9) | 26,494 (47.1) | 10,304 (68.2) | 16,940 (48.1) | 15,649 (57.2) | 31,733 (41.0) | 2,211,283 (55.9) | 3,274,796 (45.2) |
| Health facilities, number | NR | 41 | NR | 50 | NR | 72 | NR | 63 | NR | 3,981 |
| Health facilities / 10,000 persons | NR | 4.0 | NR | 3.7 | NR | 8.6 | NR | 3.6 | NR | 2.7 |

^a^Limited to adults aged 25 years and older

^b^Educated at high-school level or above; includes secundario, educación básica, educación media, ciclo postbachillerato, superior, and school postgrado (2010) or secundario, educación básica, and educación media (1990)

NR: not reported, SD: standard deviation, pct: percentile

**Supplementary Table 4.** Overall population, total cancer-related deaths, and overall cancer mortality rates in the northern Amazon provinces of Ecuador, 1990, 2001, and 2010

|  |  | Year | | | | | | | | | | | |
| --- | --- | --- | --- | --- | --- | --- | --- | --- | --- | --- | --- | --- | --- |
|  |  | 1990 | | | 2001 | | | 2010 | | | 1990-2010 | | |
| Province | Canton | Population | Cancer Deaths | Cancer Death Rate^a^ | Population | Cancer Deaths | Cancer Death Rate^a^ | Population | Cancer Deaths | Cancer Death Rate^a^ | Population | Cancer Deaths | Cancer Death Rate^a^ |
| **Oil-producing area** | |  |  |  |  |  |  |  |  |  |  |  |  |
| Sucumbíos | Lago Agrio | 37,732 | 5 | 13.3 | 66,788 | 6 | 9.0 | 91,744 | 19 | 20.7 | 1,393,988 | 245 | 17.6 |
|  | Putumayo | 4,797 | 1 | 20.8 | 6,171 | 1 | 16.2 | 10,174 | 0 | 0.0 | 136,722 | 10 | 7.3 |
|  | Shushufindi | 18,977 | 5 | 26.3 | 32,184 | 7 | 21.7 | 44,328 | 8 | 18.0 | 654,658 | 120 | 18.3 |
|  | Cascales | 5,014 | 0 | 0.0 | 7,409 | 1 | 13.5 | 11,104 | 2 | 18.0 | 158,687 | 28 | 17.6 |
|  | Cuyabeno | 3,522 | 0 | 0.0 | 6,643 | 1 | 15.1 | 7,133 | 1 | 14.0 | 115,009 | 11 | 9.6 |
| Orellana | Francisco de Orellana | 19,674 | 3 | 15.2 | 42,010 | 8 | 19.0 | 72,795 | 3 | 4.1 | 902,464 | 141 | 15.6 |
|  | Joya de los Sachas | 16,193 | 3 | 18.5 | 26,363 | 9 | 34.1 | 37,591 | 7 | 18.6 | 534,250 | 127 | 23.8 |
| Total |  | 105,909 | 17 | 16.1 | 187,568 | 33 | 17.6 | 274,869 | 40 | 14.6 | 3,895,778 | 682 | 17.5 |
|  | |  |  |  |  |  |  |  |  |  |  |  |  |
| **Non-oil-producing area** | |  |  |  |  |  |  |  |  |  |  |  |  |
| Napo | Tena | 33,967 | 9 | 26.5 | 46,007 | 4 | 8.7 | 60,880 | 16 | 26.3 | 965,589 | 148 | 15.3 |
|  | Archidona | 12,258 | 1 | 8.2 | 18,551 | 4 | 21.6 | 24,969 | 3 | 12.0 | 389,318 | 57 | 14.6 |
|  | El Chaco | 4,445 | 0 | 0.0 | 6,133 | 1 | 16.3 | 7,960 | 2 | 25.1 | 125,195 | 39 | 31.2 |
|  | Quijos | 3,847 | 3 | 78.0 | 5,505 | 1 | 18.2 | 6,224 | 3 | 48.2 | 103,875 | 43 | 41.4 |
|  | Carlos Julio Arosemen Tola | 1,780 | 0 | 0.0 | 2,943 | 1 | 34.0 | 3,664 | 0 | 0.0 | 57,045 | 9 | 15.8 |
| Pastaza | Pastaza | 29,780 | 13 | 43.7 | 45,512 | 23 | 50.5 | 62,016 | 27 | 43.5 | 936,301 | 430 | 45.9 |
|  | Mera | 5,947 | 1 | 16.8 | 8,088 | 3 | 37.1 | 11,861 | 5 | 42.2 | 165,373 | 71 | 42.9 |
|  | Santa Clara | 2,497 | 0 | 0.0 | 3,029 | 1 | 33.0 | 3,565 | 1 | 28.1 | 61,698 | 15 | 24.3 |
|  | Arajuno | 3,594 | 0 | 0.0 | 5,150 | 0 | 0.0 | 6,495 | 0 | 0.0 | 102,852 | 3 | 2.9 |
| Sucumbíos | Gonzalo Pizarro | 4,472 | 1 | 22.4 | 6,964 | 0 | 0.0 | 8,599 | 0 | 0.0 | 133,667 | 10 | 7.5 |
|  | Sucumbíos | 2,441 | 1 | 41.0 | 2,836 | 1 | 35.3 | 3,390 | 4 | 118.0 | 60,522 | 17 | 28.1 |
| Orellana | Aguarico | 3,150 | 0 | 0.0 | 4,658 | 0 | 0.0 | 4,847 | 0 | 0.0 | 85,372 | 20 | 23.4 |
|  | Loreto | 8,073 | 0 | 0.0 | 13,462 | 0 | 0.0 | 21,163 | 1 | 4.7 | 283,837 | 10 | 3.5 |
| Total |  | 116,251 | 29 | 24.9 | 168,838 | 39 | 23.1 | 225,633 | 62 | 27.5 | 3,470,644 | 872 | 25.1 |

^a^per 100,000

1. As an example, Arajuno and Curaray Parishes in Pastaza Canton were split out in 1998 to form the new canton of Arajuno. Therefore, the population reported for Pastaza Canton in the 1990 census included persons living in the future Arajuno Canton, whereas the population of Pastaza in the 2001 and 2010 censuses did not. To eliminate this inconsistency, we identified census records from Arajuno and Curaray Parishes in the 1990 census as belonging to the future Arajuno Canton. As a result, Pastaza and Arajuno Cantons referred to two distinct and consistent geographic areas throughout the entire study period. [↑](#footnote-ref-1)
